# Overexpression of the *CmJAZ1-like* gene delays flowering in *Chrysanthemum morifolium*

**DOI:** 10.1038/s41438-021-00525-y

**Published:** 2021-04-01

**Authors:** Yunxiao Guan, Lian Ding, Jiafu Jiang, Yuanyue Shentu, Wenqian Zhao, Kunkun Zhao, Xue Zhang, Aiping Song, Sumei Chen, Fadi Chen

**Affiliations:** State Key Laboratory of Crop Genetics and Germplasm Enhancement, Key Laboratory of Landscaping, Ministry of Agriculture and Rural Affairs, College of Horticulture, Nanjing Agricultural University, Nanjing, 210095 China

**Keywords:** Transgenic plants, Flowering

## Abstract

Chrysanthemum (*Chrysanthemum morifolium*) is one of the four major cut-flower plants worldwide and possesses both high ornamental value and cultural connotation. As most chrysanthemum varieties flower in autumn, it is costly to achieve annual production. *JAZ* genes in the TIFY family are core components of the jasmonic acid (JA) signaling pathway; in addition to playing a pivotal role in plant responses to defense, they are also widely implicated in regulating plant development processes. Here, we characterized the TIFY family gene *CmJAZ1-like* from the chrysanthemum cultivar ‘Jinba’. CmJAZ1-like localizes in the nucleus and has no transcriptional activity in yeast. Tissue expression pattern analysis indicated that *CmJAZ1-like* was most active in the root and shoot apex. Overexpressing *CmJAZ1-like* with Jas domain deletion in chrysanthemum resulted in late flowering. RNA-Seq analysis of the overexpression lines revealed some differentially expressed genes (DEGs) involved in flowering, such as the homologs of the flowering integrators *FT* and *SOC1*, an FUL homolog involved in flower meristem identity, AP2 domain-containing transcription factors, MADS box genes, and autonomous pathway-related genes. Based on KEGG pathway enrichment analysis, the differentially transcribed genes were enriched in carbohydrate metabolic and fatty acid-related pathways, which are notable for their role in flowering in plants. This study preliminarily verified the function of *CmJAZ1-like* in chrysanthemum flowering, and the results can be used in molecular breeding programs aimed at flowering time regulation of chrysanthemum.

## Introduction

Thines et al.^1^ performed exogenous jasmonic acid (JA) treatment of the *Arabidopsis* mutant *opr3*, and eight proteins containing the conserved ZIM domain were significantly induced, which led to the discovery of JAZ repressor proteins^1,2^. JAZs belong to the plant-specific TIFY family and possess three conserved domains: NT, ZIM, and Jas^3^. The N-terminus of the protein contains a weakly conserved NT domain, which can interact with the DELLA protein and the flowering repressor TARGET OF EAT1 (TOE1)^4,5^. The ZIM domain consists of 36 amino acids, including the conserved TIFY motif (TIF [F/Y] XG), which is essential for the formation of homo- or heterodimers among JAZ proteins and determines the combination of JAZ and NINJA in the JAZ-NINJA-TPL repressor complex^6,7^. The C-terminal Jas domain is highly conserved, consisting of 12–29 amino acids, and controls interactions with the F-box protein CORONATINE INSENSITIVE1 (COI1)^8,9^. The function of JAZs in the JA signaling pathway has been revealed in *Arabidopsis*. The F-box protein COI1 perceives JA-Ile and forms the E3 ubiquitin ligase SCF^COI1^, which further targets jasmonate-ZIM domain (JAZ) proteins for degradation through the 26S proteasome. Consequently, the transcription factors that are bound and repressed by JAZs are released, thereby regulating plant developmental processes and defense responses^1,10,11^.

Flowering at the suitable time is crucial for prosperous reproduction and has important commercial value for ornamental plants. The transformation of flowering plants from vegetative growth to reproductive growth is coordinated by a series of signal transduction pathways and sophisticated gene networks. Thus far, it has been proven that the photoperiod, vernalization, gibberellin, aging, autonomous, and ambient temperature pathways are involved in regulating the flowering time of *Arabidopsis*^12^. Among them, the autonomous, age, and gibberellin pathways regulate flowering time through endogenous signals, which are closely related to the growth and development status of the plant itself, whereas the photoperiod and vernalization pathways respond to external environmental stimuli^13^. These six predominant pathways ultimately gather the common downstream floral integrators FLOWERING LOCUS T (FT) and SUPPRESSOR OF OVEREXPRESSION OF CO1 (SOC1), which can activate the expression of floral meristem identity genes, such as *APETALA1* (*AP1*), *LEAFY* (*LFY*), and *FRUITFULL* (*FUL*), and consequently lead to flowering^14^.

As pivotal components of the JA signaling pathway, JAZs not only play a crucial role in plant responses to environmental stresses and biotic challenges^10,15,16^ but also are widely implicated in the regulation of plant development processes, such as root growth^17,18^, leaf senescence^19^, trichome initiation^20^, anthocyanin accumulation^20^, flower abscission^21^, stamen development^22^, spikelet development^23^, and seed production^24^. To date, few studies have reported the relationship between JAZs and flowering regulation. Zhai et al.^5^ revealed that JAZ proteins (JAZ1, JAZ3, JAZ4, and JAZ9) could interact with the AP2 family proteins TOE1 and TOE2 and confirmed that JAZ1 can reduce the transcriptional inhibitory effect of TOE1 on *FT* through interaction. Consequently, plants with *AtJAZ1*Δ*Jas* overexpression exhibited early flowering^5^. The *jaz7-1D* mutant acquired by T-DNA insertion of the *JAZ7* gene in the promoter led to earlier flowering than that in wild-type plants under short-day conditions^25^. Oblessuc et al.^26^ found that the mutant *jaz4-1* exhibited delayed flowering and that overexpression of the JAZ4 protein, which deletes the Jas domain, accelerated flowering compared to that in the wild type. In addition to the differences between the wild type and transgenic plants in leaf initiation, plant height, and trichomes, overexpression of *SlJAZ2* in tomato also led to advanced flowering transition^27^.

Chrysanthemum (*Chrysanthemum morifolium*) is a popular ornamental plant with high commercial value worldwide, and most chrysanthemum varieties flower in autumn. Therefore, it is essential to conduct research related to the regulation of chrysanthemum flowering time to achieve annual production. Although the role of JAZ proteins has been extensively studied in *Arabidopsis*, to the best of our knowledge, their functions in chrysanthemum have not been characterized. In this study, we isolated and characterized a TIFY family gene, *CmJAZ1-like*. The transgenic lines overexpressing *CmJAZ1-like* without the Jas domain delayed flowering in *C. morifolium*. RNA-Seq analysis indicated that genes associated with flowering were differentially expressed. KEGG pathway enrichment analysis showed that a total of 1463 differentially expressed genes (DEGs) were enriched in carbohydrate metabolism and fatty acid-related pathways that are crucial for floral induction in plants^28,29^. Taken together, the results of this study link *CmJAZ1-like* to flowering time through regulation of the genes related to flowering and the metabolic processes of carbohydrates and fatty acids in chrysanthemum.

## Results

### Isolation and sequence analyses of CmJAZ1-like

**Fig. 1:**
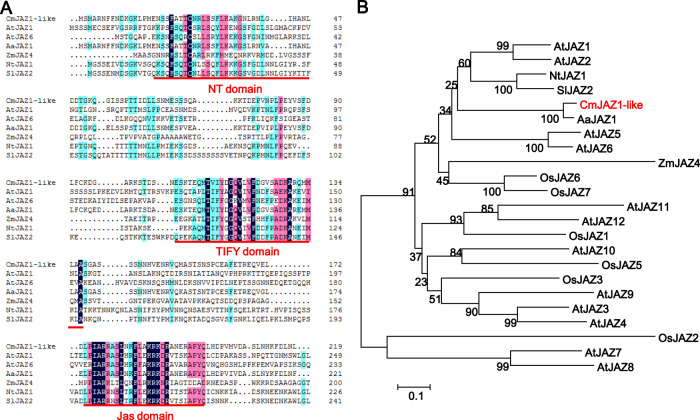
Amino acid sequence analysis and phylogenetic tree of JAZ proteins. **A** Polypeptide sequence alignment of CmJAZ1-like and homologous proteins from other species. Navy blue represents 100% identity, pink represents 75% identity, and light blue represents 50% identity. **B** Phylogenetic tree of CmJAZ1-like and related JAZ proteins from other species using the neighbor-joining method. The bar (0.1) indicates the branch length. The gene accession numbers are as follows: *Arabidopsis thaliana AtJAZ1* (At1g19180), *AtJAZ2* (At1g74950), *AtJAZ3* (At3g17860), *AtJAZ4* (At1g48500), *AtJAZ5* (At1g17380), *AtJAZ6* (At1g72450), *AtJAZ7* (At2g34600), *AtJAZ8* (At1g30135), *AtJAZ9* (At1g70700), *AtJAZ10* (At5g13220), *AtJAZ11* (At3g43440), *AtJAZ12* (At5g20900), *Oryza sativa OsJAZ1* (XP_015635690.1), *OsJAZ3* (XP_015651050.1), *OsJAZ2* (XP_015646242.1), *OsJAZ5* (XP_015634258.1), *OsJAZ6* (XP_015630632.1), *OsJAZ7* (XP_015647536.1), *Artemisia annua AaJAZ1* (PWA58763.1), *Zea mays ZmJAZ4* (XP_008664988), *Nicotiana tabacum NtJAZ1* (BAG68655.1), and *Solanum lycopersicum SlJAZ2* (NP_001234883.1)

As a gene encoding a protein that interacts with the positive regulator of chrysanthemum petal elongation CmTCP20, *CmJAZ1-like* was previously isolated from ‘Jinba’ chrysanthemum^30^ and comprises a 657-bp open reading frame (ORF) that encodes a 219-amino-acid polypeptide. CmJAZ1-like belongs to the TIFY protein family and has three conserved domains. As shown in Fig. 1A, the first conserved domain, the NT domain, is located at the N-terminus and exhibits weak conservation; the second is the TIFY domain, which is between 313 and 411 bp in size; and the last is the Jas domain, which is strongly conserved between 523 and 597 bp and is crucial for SCF^COI1^-mediated degradation of JAZ^1,10^. The obtained phylogenetic tree revealed that CmJAZ1-like was highly homologous with the *Artemisia annua* AaJAZ1 protein (Fig. 1B), so this gene was named *CmJAZ1-like*. Amino acid sequence alignment indicated that the amino acid sequence similarity between CmJAZ1-like and homologous proteins in other species was 87.78% (AaJAZ1), 30.95% (NtJAZ1), 30.04% (AtJAZ1), 19.07% (ZmJAZ4), and 31.50% (SlJAZ2).

### Subcellular localization, transcriptional activation, and expression patterns of CmJAZ1-like

**Fig. 2:**
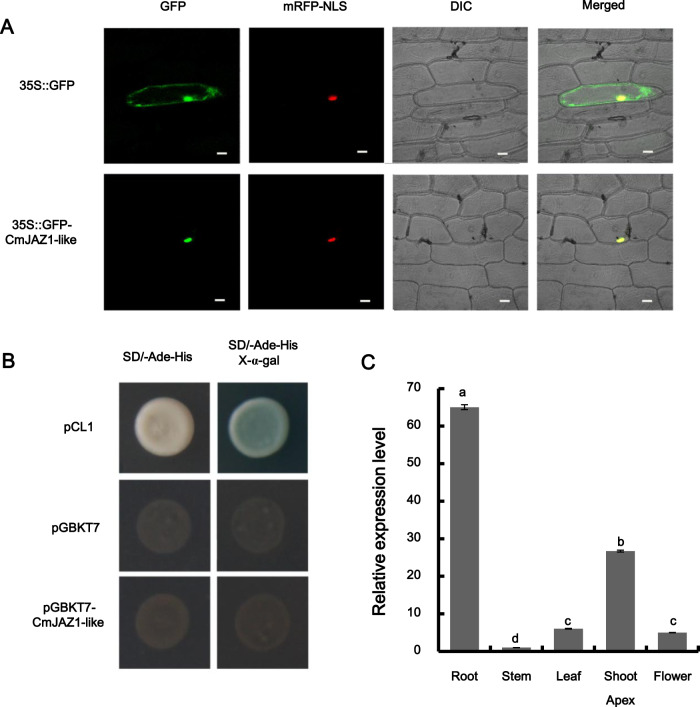
Subcellular localization, transactivation, and expression pattern analysis of CmJAZ1-like. **A** Subcellular localization of CmJAZ1-like in onion cells. The coexpressed *35S::D53-RFP* construct was used as a nuclear marker. Bar: 50 μm. **B** Transcriptional activation of CmJAZ1-like in yeast cells. pCL1 was the positive control, and pGBKT7 was the negative control. Left: SD/-Ade-His medium, right: SD/-Ade-His medium coated with X-α-gal. **C** Expression pattern of *CmJAZ1-like* in various organs of the wild-type chrysanthemum cultivar ‘Jinba’. *CmEF1α* (GenBank: AB548817.1) was used as an endogenous control in chrysanthemum. The 2^−ΔΔCt^ method was used to calculate relative transcript abundances. The values are presented as the mean ± SE (*n* = 3). Significant differences were analyzed using Duncan’s multiple-range test. (*P* < 0.05)

To investigate the subcellular localization of CmJAZ1-like, the control vector *35S::GFP* and the constructed vector *35S::GFP-CmJAZ1-like* were introduced into onion epidermal cells via particle bombardment. In the transformed cells, red fluorescent protein was utilized as the nuclear marker (D53-mCherry); the green fluorescent protein (GFP) fluorescence of the control vector was observed in both the cytoplasm and the nucleus, while the GFP fluorescence of the constructed vector *35S::GFP-CmJAZ1-like* was detected in only the nucleus of the onion epidermal cells (Fig. 2A). These results suggested that CmJAZ1-like localizes in the nucleus.

To further examine the transcriptional activation of CmJAZ1-like, the gene was fused to the GAL4-binding domain (BD) and expressed in the yeast strain Y2H. The pCL1 vector served as the positive control, while the pGBKT7 vector was the negative control. The results showed that yeast cells harboring the pCL1 vector grew extremely well on SD/-Ade-His medium and could turn blue on SD/-Ade-His medium coated with X-α-gal, whereas those harboring the pGBKT7 vector and the constructed pGBKT7-CmJAZ1-like vectors were unable to grow on SD/-Ade-His medium, which suggested that CmJAZ1-like exhibited no transcriptional activation (Fig. 2B).

The expression pattern of *CmJAZ1-like* in different tissues of ‘Jinba’ chrysanthemum plants was determined via qRT-PCR. The data showed that *CmJAZ1-like* was most abundantly transcribed in the root, followed by the shoot apex, leaf, and flower, with only a low level of transcript detectable in the stem (Fig. 2C).

### Overexpression of *CmJAZ1-like*Δ*Jas* delays flowering in *C. morifolium*

**Fig. 3:**
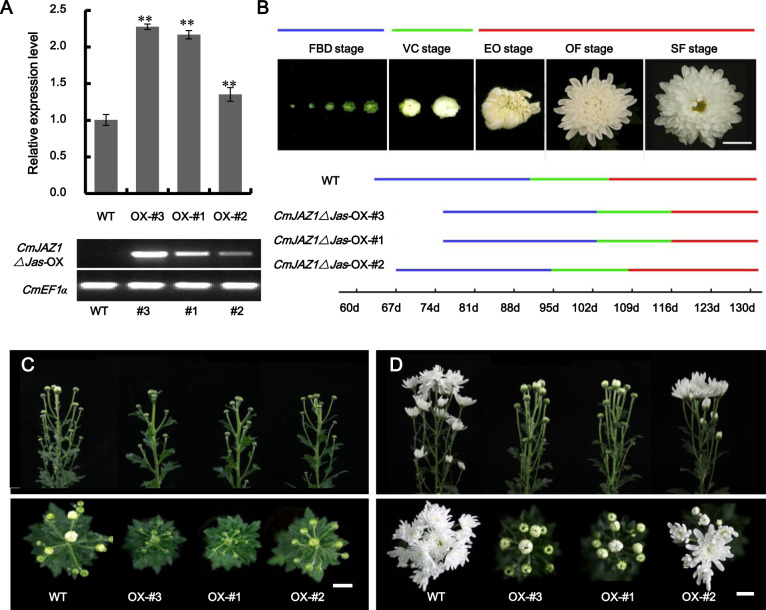
Identification and phenotypes of *CmJAZ1-like*Δ*Jas*-overexpressing transgenic ‘Jinba’ plants. **A** Top figure: expression of *CmJAZ1-like* in the wild-type and transgenic plants, as determined by qRT-PCR. OX-#3, OX-#1, and OX-#2 represent three independent *CmJAZ1-like*Δ*Jas*-overexpressing lines. Significant differences were determined by Student’s *t* test (**P < 0.01). Bottom figure: RT-PCR analysis of *CmJAZ1-like* in the wild-type and transgenic lines at the cDNA level. *CmEF1α* is an endogenous control. **B** Developmental process of flower buds in the wild-type and transgenic plants. *FBD* flower bud development stage, *VC* visible color stage, *EO* earlier opening stage, *OF* open-flower stage, *SF* senescent-flower stage. Bars: 2 cm. **C**, **D** Phenotypes of chrysanthemum-overexpressing lines at the reproductive stage. Photographs were acquired at 91 days (**C**) and 104 days (**D**) after transplantation

The Jas domain of JAZ proteins is essential for SCF^COI1^-dependent proteasome degradation through interactions with COI1^1,31^. Overexpression of the sequence with Jas domain deletion or mutation has been widely used to study the function of *JAZ* genes^5,26,32^. To further investigate the biological function of *CmJAZ1-like* in chrysanthemum, we transformed the CaMV 35S promoter followed by the coding sequence (CDS) of *CmJAZ1-like* without the Jas domain into ‘Jinba’ chrysanthemum through *Agrobacterium*-mediated leaf disc transformation, obtaining eight *35Spro:CmJAZ1-like*Δ*Jas* overexpression lines that were validated by PCR at the DNA level and by qRT-PCR analysis during the tissue culture period (Fig. S1). We then transplanted three representative *CmJAZ1*Δ*Jas-*overexpressing transformants (OX-#3, OX-#1, and OX-#2) to the field for further phenotypic observation. These three positive transgenic lines were reconfirmed through RT-PCR analysis by using specific primers designed using the GFP tag as a forward primer and the gene CDS as a reverse primer, and their transcriptional levels were measured by qRT-PCR using *CmJAZ1-like*-specific primers (Fig. 3A).

We observed that the flower buds of transgenic plants emerged later than those of the wild-type plants under natural conditions. At 63 d after transplantation, flower buds were found in most of the wild-type plants, while this did not occur in the *CmJAZ1*Δ*Jas*-OX-#3 and *CmJAZ1*Δ*Jas*-OX-#1 plants. Moreover, at 91 d after transplantation, the wild-type plants had entered the visible color stage, while the *CmJAZ1*Δ*Jas*-OX-#3 and *CmJAZ1*Δ*Jas*-OX-#1 plants were still at the flower bud development stage (Fig. 3B, C), and at 104 d after planting, the wild-type plants were already exhibiting flower opening, while the *CmJAZ1*Δ*Jas*-OX-#3 and *CmJAZ1*Δ*Jas*-OX-#1 plants were at the visible color stage (Fig. 3D). Subsequently, the time when flower buds emerged and bloomed in the *CmJAZ1*Δ*Jas*-OX-#3 and *CmJAZ1*Δ*Jas*-OX-#1 plants was 12 d later than that in the wild-type plants. Furthermore, the difference in flowering time observed between *CmJAZ1*Δ*Jas*-OX-#2 and the wild-type plants was not as apparent (only 4 d). The severity of the phenotype was positively correlated with the expression level of the transgenic lines. These results indicate that *CmJAZ1-like* has the capacity to regulate flowering time in chrysanthemum.

### Transcriptome sequencing analysis and functional enrichment of DEGs in *CmJAZ1-like*Δ*Jas* overexpression lines

**Fig. 4:**
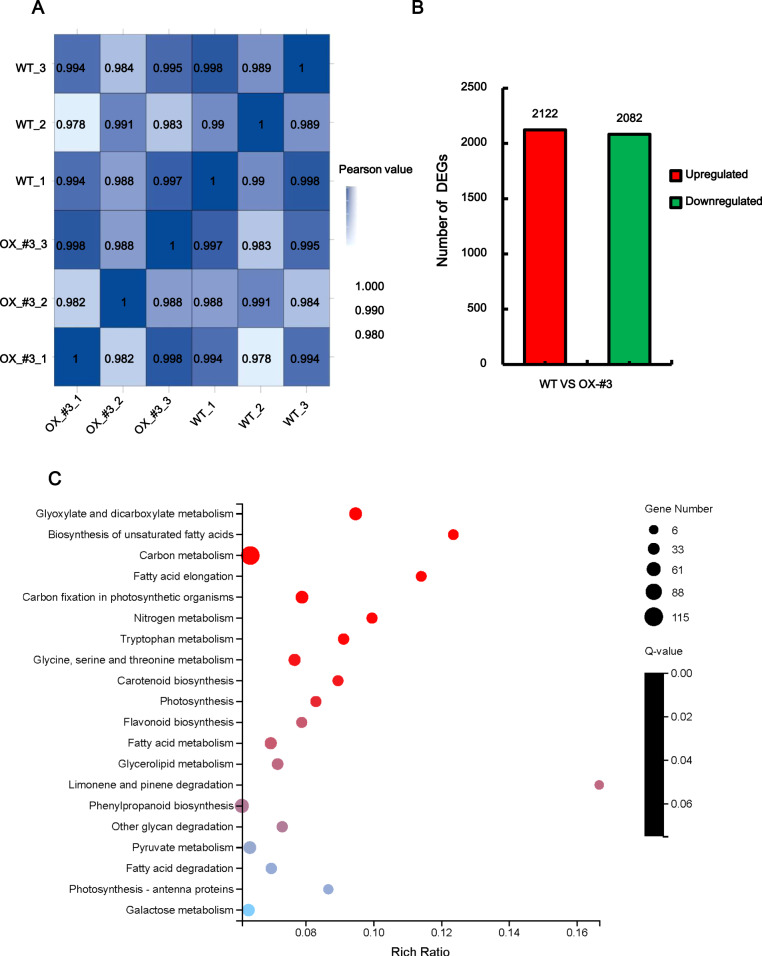
Global analysis of the transcriptome data and DEGs analysis of the wild-type and *CmJAZ1-like*Δ*Jas*-overexpressing transgenic plants. **A** Pearson’s correlation between six samples. **B** Number of up- or downregulated genes in the comparison between the wild-type and *CmJAZ1*Δ*Jas*-OX-3# transgenic plants. **C** KEGG pathway enrichment of 1463 DEGs with KEGG annotations. The *X*-axis represents the enrichment ratio (Rich Ratio = Term Candidate Gene Num / Term Gene Num), the *Y*-axis represents KEGG pathways, the size of the bubble represents the number of genes annotated to a KEGG pathway, and the color represents the enrichment Q-value. The darker the color is, the smaller the Q-value

To better understand the regulatory mechanisms of *CmJAZ1-like* involved in the regulation of flowering time, RNA-seq analysis was performed. RNA extracted from the seventh unfolded leaf of the wild-type and *CmJAZ1*Δ*Jas*-OX-#3 plants was used as the RNA-Seq sample. A total of 390.5 M clean reads were generated from six samples (three replicates each for the wild-type and transgenic lines), with each sample producing a minimum of 62.66 M clean reads. After implementing the assembly procedure, we obtained 111,669 unigene sequences with a mean length of 1159 bp; the N50 was 1703 bp (Table S1). The Pearson’s correlation coefficient of the three wild-type samples ranged from 0.989 to 1, and that of the *CmJAZ1*Δ*Jas-*OX-#3 samples ranged from 0.982 to 1 (Fig. 4A), which indicated that the transcript abundances of the biological replicate samples were highly correlated. The DEseq2 method was used to identify DEGs^33^. A total of 4204 DEGs were obtained between the wild-type and *CmJAZ1*Δ*Jas*-OX-#3 with a Q value (adjusted *P* value) ≤ 0.05 as the standard. Among these genes, 2122 were upregulated, and 2082 were downregulated (Fig. 4B; Table S3).

Among the 4204 DEGs, a total of 1463 possessed KEGG annotations. To explore the functional categories and main biological pathways involving these DEGs, according to KEGG pathway annotation classification, we used the phyper function in R software for enrichment analysis and selected the 20 KEGG terms with the lowest Q values as a showcase (Fig. 4C). Most of these transcripts were involved in metabolic pathways. These metabolic pathways included five carbohydrate metabolic pathways and five fatty acid-related pathways. Carbohydrates play a crucial role in floral induction as the signal and energy supply factors^34,35^. In our study, a maximum of 115 genes were annotated to carbon metabolism, and a total of 47 candidate genes were enriched to glyoxylate and dicarboxylate metabolism. Moreover, 45 genes were involved in carbon fixation in photosynthetic organisms; the glycan degradation and galactose metabolism KEGG terms contained 28 and 42 genes, respectively. In addition, five fatty acid-related pathways, including “Biosynthesis of unsaturated fatty acids”, “Fatty acid elongation”, “Fatty acid metabolism”, “Glycerolipid metabolism”, and “Fatty acid degradation”, were also enriched. It has been reported that fatty acids are involved in flowering regulation^36^. These results indicate that carbohydrates and fatty acids may be the cause of the difference in flowering time between the transgenic and wild-type plants. Pathways such as “Tryptophan metabolism”, “Glycine, serine, and threonine metabolism”, “Photosynthesis”, and “Photosynthesis-antenna proteins”, which are related to flowering, were also represented. In addition to the difference in flowering time, we also observed growth-restricted roots and smaller leaves in the *CmJAZ1-like*Δ*Jas* overexpression plants compared to the wild type. These may also be the result of changes in metabolic pathway-related genes.

### Flowering-related genes were differentially expressed in *CmJAZ1-like*Δ*Jas* overexpression lines

Given that overexpression of *CmJAZ1-like* with the Jas domain deleted delays flowering, we focused on the DEGs between *CmJAZ1*Δ*Jas*-OX-#3 and wild-type plants that were implicated in the flowering pathways (Table 1). According to the annotation, we found that the homologs of the flowering integrators *FT* (CL3262.Contig5_All) and *SOC1* (CL2913.Contig4_All) were significantly decreased in the *CmJAZ1*Δ*Jas*-OX-#3 lines. *FUL* has been reported as a positive regulator of flower meristem identity^37^, and its homologous gene (CL10079.Contig1_All) was also downregulated, whereas homologs of the MADS box genes *FLC* (CL14305.Contig7_All) and *SVP* (CL7056.Contig2_All), which act as flowering inhibitors, were upregulated in *CmJAZ1*Δ*Jas*-OX-#3 compared to that in the wild-type strain. In addition, two AP2 domain-containing transcription factors, namely, *CmTOE3* (Unigene26493_All) and *CmTEM1* (Unigene36566_All), homologs of which in *Arabidopsis* can repress the expression of the florigen *FT*^38^, were also more abundant in *CmJAZ1*Δ*Jas*-OX-#3. Notably, the homologs of *DRM1* (CL4634.Contig9_All) and *FVE* (CL1053.Contig1_All), which are involved in autonomous pathways, were significantly reduced. We also observed that the homologous genes of the rhythm regulators *RVE1* (CL2797.Contig1_All) and *ELF4* (Unigene1508_All) were mildly altered.

**Table 1:** DEGs related to flowering time between the wild-type and *CmJAZ1-like*Δ*Jas*-overexpressing transgenic plants

Gene_ID	Annotation	log2(OX-JAZ/WT)	Q-value	Function
CL2913.Contig4_All	SOC1	−2.41	0.03	Flowering integrator
CL3262.Contig5_All	FTL	−1.37	8.42E-14	Flowering integrator
Unigene36566_All	TEM1	0.55	0.009	AP2 domain transcription factor
Unigene26493_All	TOE3	5.40	2.38E-24	AP2 domain transcription factor
CL14305.Contig7_All	FLC	1.15	0.016	MADS box gene
CL7056.Contig2_All	SVP	1.24	6.95E-07	MADS box gene
CL10079.Contig1_All	FUL	−1.35	4.93E-12	MADS box gene
CL2797.Contig1_All	RVE1	−0.67	0.0003	Photoperiod pathway
Unigene1508_All	ELF4	0.59	0.009	Photoperiod pathway
CL4634.Contig9_All	DRM1	−2.24	0.0006	Autonomous pathway
CL1053.Contig1_All	FVE	−2.78	5.03E-26	Autonomous pathway

Significant differences were determined with Q < 0.05 and |log2(OX-JAZ/WT) | > 0.5

*WT* wild type

**Fig. 5:**
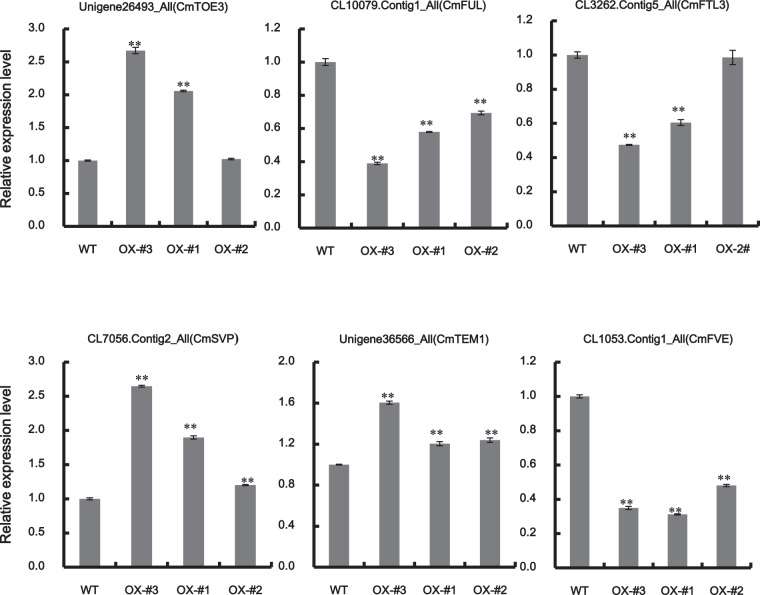
qRT-PCR validation of the genes selected from DEGs involved in flowering regulation. *CmEF1α* (GenBank: AB548817.1) was used as an endogenous control in chrysanthemum. The values are presented as the mean ± SE (*n* = 3). Significant differences were determined by Student’s *t* test (**P < 0.01)

To verify the authenticity of the expression levels obtained from the transcriptome data, we selected some of the genes related to flowering time above and detected their transcripts using real-time quantitative PCR. In ‘Jinba’ chrysanthemum, the expression of the *elongation factor 1α* (*CmEF1α*) gene was used as an internal control. The templates for qRT-PCR were sourced from the seventh unfolded leaf of the wild type and the transgenic lines *CmJAZ1*Δ*Jas*-OX-#3, *CmJAZ1*Δ*Jas*-OX-#1, and *CmJAZ1*Δ*Jas*-OX-#2. As shown in Fig. 5, the changes in transcript expression obtained by qRT-PCR were identical to those acquired by DEG expression profiling. Collectively, the above expression results suggest that the transcriptome data were credible and that *CmJAZ1-like* affects flowering time by regulating the genes related to flowering.

## Discussion

As essential components of JA signal transduction, JAZ proteins not only inhibit the transcription of the JA signaling response but also interact with and repress other transcription factors to affect various signaling pathways and metabolic processes of plant growth^39^. To the best of our knowledge, there have been no studies on the function of TIFY family genes in chrysanthemum. Here, we identified the TIFY gene *CmJAZ1-like*, and transcription profiling indicated that *CmJAZ1-like* was abundantly expressed in the root and shoot apex. While expression in the shoot apex may be involved in flowering time control (Fig. 2C), the most active expression was observed in the root, which may reveal an additional function of *CmJAZ1-like*. Phylogenetic analysis and amino acid sequence alignment indicated that the CmJAZ1-like protein is closely related to another composite protein, AaJAZ1, with 87.78% sequence similarity, followed by the SlJAZ2 and AtJAZ1 proteins in another branch, the sequence similarities of which with CmJAZ1-like were only 31.50% and 30.04%, respectively (Fig. 1A, B). This revealed that the sequence and structure of JAZ proteins in different species listed in the phylogenetic tree, except *Artemisia annua*, displayed marked differences.

Flowering is a very complex process that is affected by endogenous developmental signals and external environmental factors^13^. Based on RNA-seq transcript profiling, we obtained 12 flowering-related homologous genes that were differentially expressed in *CmJAZ1-like*Δ*Jas-*overexpressing transgenic chrysanthemum plants compared with wild-type plants (Table 1). *FUL* is redundant with *AP1* in regulating floral meristem identity^40^ and is also implicated in flowering^41^. Zhao et al.^42^ generated *p35S::GFP-CmFL2* transgenic chrysanthemum plants that exhibited early flowering. *CmFUL* is a homologous gene of *CmFL2* and was significantly downregulated in *CmJAZ1-like*Δ*Jas* transgenic plants. The transcription factors TEM1 and TOE3 containing the AP2 domain are repressors of the florigen *FT*. Zhai et al.^5^ revealed that JAZ1 could interact with TOE1 and thereby reduce the transcriptional inhibitory effect of TOE1 on *FT*, leading to early flowering. In this study, the expression levels of *CmTOE3* and *CmTEM1* were upregulated, whereas *CmFTL3* displayed decreased expression. These results indicated that *CmJAZ1-like* can influence the AP2 domain transcription factors *CmTOE3* and *CmTEM1* at the transcriptional level in chrysanthemum. However, the regulatory mechanism between them requires further investigation. The autonomous pathway is an independent method for induction of flower formation that is not regulated by external signals. The autonomous pathway genes regulate *FT* by repressing *FLC* and *SVP*. FVE is a key regulator in this pathway; it binds to CLF-PRC2 via the encoded WD40 protein MSI4 to inhibit *FLC* expression^43^. Zhu et al.^44^ confirmed that *drm1* is a typical late flowering mutant and is most likely associated with the autonomous flowering pathway. Moreover, *FLC* had lower expression in *drm1*. As shown in Table 1, the expression levels of the *CmDRM1* and *CmFVE* genes were significantly reduced, whereas those of *CmFLC* and *CmSVP* were highly increased, in *CmJAZ1*Δ*Jas*-OX-#3. These results showed that *CmJAZ1-like-*mediated regulation of flowering time depends partly on the autonomous pathway. Among these DEGs, the homologous genes of the rhythm regulators *RVE1* and *ELF4*, which are involved in the photoperiod pathway^45^, were mildly altered at the transcription level. Therefore, overexpression of the *CmJAZ1-like* gene caused increased expression of the AP2 transcription factors *CmTOE3* and *CmTEM1*, reduced expression of autonomous pathway genes, and slight changes in the rhythm regulators *CmRVE1* and *CmELF4*. All of these factors together induced the expression of the flowering inhibitors *CmFLC* and *CmSVP*, in addition to downregulating the integrators *CmFTL3* and *CmSOC1* and a positive regulator of flower meristem identity, resulting in delayed flowering^46,47^.

Previous studies have demonstrated that carbohydrates and fatty acids are essential for floral induction in plants^28,29^. Through KEGG pathway enrichment analysis, the differentially transcribed genes between the transgenic and wild-type plants were enriched in carbohydrate metabolic and fatty acid-related pathways. Carbohydrates such as sucrose, glucose, and starch can provide energy for flower induction and are important signal transmission factors during this process^34,35^. The effect of fatty acids on flowering regulation has also been confirmed. In *Arabidopsis*, overexpressing a fatty acid amide hydrolase gene can accelerate flowering^36^. Furthermore, in a de novo transcriptome study of *Dendrobium*, a total of 84 flower-specific expressed transcripts were also enriched in two fatty acid biosynthesis-related pathways^48^. Chen et al.^49^ reported that DEGs between two apple varieties with different flowering abilities were mainly involved in carbohydrate, fatty acid, and lipid pathways. This indicated that the delay in flowering caused by overexpressing *CmJAZ1-like* may be ascribed to the changes in the carbohydrate and fatty acid pathways.

In the present study, overexpression of *CmJAZ1-like*Δ*Jas* delayed flowering in *C. morifolium*. However, in *Arabidopsis*, *AtJAZ1*Δ*Jas* overexpression in plants accelerated flowering compared to that in the wild type^5^. A similar situation was also observed in a study of the tomato *SlJAZ2* gene, which is homologous to *AtJAZ1*^27^. In *Arabidopsis*, ectopic expression of the full-length JAZ1 cDNA did not lead to an early flowering phenotype; however, *35Spro:SlJAZ2* transgenic plants flowered one week earlier than the wild type^27^. Furthermore, with overexpression of the R2R3 MYB transcription factor *CmMYB2* in *Arabidopsis* and chrysanthemum, the transgenic plants exhibited an opposite phenotype in terms of flowering time^50^. These phenomena probably result from the differences in the sequence and structure of proteins in different species, which generate diverse protein characteristics and lead to the formation of different dimers with different proteins, which further participate in diverse regulatory pathways. In addition, *Arabidopsis* is a long-day plant, whereas the ‘Jinba’ chrysanthemum is a short-day plant. The photoperiod conditions and different day-length requirements for flowering may also be the cause of these discrepant phenotypes. However, the detailed mechanism leading to these differences requires further research.

In summary, we isolated a TIFY gene, *CmJAZ1-like*, from chrysanthemum and found clues that *CmJAZ1-like* functions in the chrysanthemum flowering process. Transcriptome sequencing revealed that flowering inhibitors, such as homologs of *FLC*, *SVP*, and AP2 domain-containing transcription factors, were upregulated, while homologs of the flowering integrators *FT* and *SOC1*, one *FUL* homolog, and homologs of the autonomous pathway genes *DRM1* and *FVE* were significantly downregulated. A total of 1463 DEGs with KEGG annotations were enriched in carbohydrate metabolic and fatty acid-related pathways. This study identified the function of *CmJAZ1-like* in the flowering regulation of chrysanthemum, laying a foundation for developing molecular breeding programs aimed at flowering time regulation of chrysanthemum in the future.

## Materials and methods

### Plant materials and growth conditions

In this study, we used a popular cut-flower chrysanthemum cultivar, ‘Jinba’, with white flowers, as the genetically modified material. Cuttings of the transgenic and wild-type plants were sourced from the Chrysanthemum Germplasm Resource Preserving Center, Nanjing Agricultural University (Nanjing, China). Four- to five-leaf-stage rooted seedlings, including the transgenic lines and wild-type plants, were transplanted in a multispan greenhouse on July 25, 2019, in which the relative humidity was maintained at 70% and the day/night temperature was 34 °C/27 °C. The flowering time was observed under natural light cycle conditions, and the observation of the phenotype was repeated three times.

### Isolation of *CmJAZ1-like*

Total RNA was extracted from snap-frozen flower buds of the ‘Jinba’ cultivar using RNAiso Plus reagent (Takara Bio, Tokyo, Japan), and 1 μg of the RNA was used for reverse transcription amplification. First-strand cDNA was synthesized by M-MLV reverse transcriptase (Takara Bio). Based on the *Unigene2375* sequence in the chrysanthemum ‘Jinba’ transcriptome^51^, the primer pair CmJAZ1-F/R was designed using Primer 5.0 software (www.bbioo.com/Soft/2005/114.htm) to amplify an internal fragment of *CmJAZ1-like* (Table S2) using Phusion High-Fidelity DNA Polymerase (Thermo Fisher Scientific, Waltham, MA, USA). To check the *CmJAZ1-like* nucleotide sequence, the amplicon was inserted into the pMD19-T vector (Takara Bio) using solution I ligase (Takara Bio) for sequencing.

### Sequence analysis of CmJAZ1-like

The amino acid sequences of JAZ proteins in *Arabidopsis* were downloaded from TAIR (http://www.arabidopsis.org/); the amino acid sequences of JAZ1 homologs in other species were acquired from GenBank (ww.ncbi.nlm.nih.gov). All of these sequences (Table S4) were subjected to phylogenetic analysis using MEGA 5.0 software based on the neighbor-joining method implemented with 1000 bootstrap replicates^52^. Multiple-sequence alignment analysis of the JAZ proteins was implemented in DNAMAN software.

### Subcellular localization of CmJAZ1-like

To understand the localization of the CmJAZ1-like protein in plant cells, we used the primer pair CmJAZ1-pENTR1A-F/R (Table S2) with the restriction site to amplify the full-length ORF of the *CmJAZ1-like* gene. Then, the amplicons and the pENTR™ 1A vector (Invitrogen, Carlsbad, CA, USA) were both restricted to *Sal I* and *Not I* and ligated after digestion using solution I ligase (Takara Bio) to generate the pENTR1A-CmJAZ1-like vector. The constructs were then recombined with the pMDC43 vector via LR Clonase™ II (Invitrogen) to obtain the GFP fusion plasmid *p35S::GFP-CmJAZ1-like*. According to the Cold Spring Harbor Experiment Manual^53^, both the *35::GFP-CmJAZ1-like* and *35::GFP* plasmids were transiently introduced into onion (*Allium cepa*) epidermal cells by particle bombardment (PDS-1000; Bio-Rad Laboratories, Hercules, CA, USA). The fluorescence signal of the transformed cells was detected using a Zeiss LSM 780 confocal microscope (Carl Zeiss AG, Oberkochen, Germany) after incubation in the dark for 16 h at 22 °C on Murashige and Skoog (MS)^54^ medium.

### Transcriptional activity analysis of CmJAZ1-like

A yeast assay system (Takara Bio) was used to examine the transcriptional activity of CmJAZ1-like. An ORF of CmJAZ1-like lacking a termination codon was amplified using the primer pair CmJAZ1-BD-F/R (Table S2). Then, the amplicon and pGBKT7 vector (Invitrogen) were both digested with *EcoRI* and *BamHI*, and the PCR products were ligated using solution I ligase (Takara Bio), generating the construct pGBKT7-CmJAZ1-like. Following the manufacturer’s protocol, the plasmids pCL1 (positive control), pGBKT7 (negative control), and pGBKT7-CmJAZ1-like were transformed into the yeast strain Y2H. Transformants containing pGBKT7-CmJAZ1-like or pGBKT7 were cultured on SD/-Trp medium, whereas those containing the positive control pCL1 were cultured on SD/-Leu medium. SD medium (a minimal, synthetic, defined medium) includes carbon sources, yeast nitrogen sources without amino acids, and dropout supplements, which can be added to the minimal SD base to make a synthetic, defined medium lacking the specified nutrients. After 3 days at 30 °C, we selected single clones and transferred them onto SD/-Ade-His medium containing either 0 or 20 mg/mL X-α-gal. Similarly, after 3 days of growth, we assessed whether there were blue spots on the plates.

### *CmJAZ1-like* genetic transformation and phenotype observation

The same method as that described in the previous subsection was used to construct the vector pENTR1A-CmJAZ1-likeΔJas. We used the primer pair CmJAZ1ΔJas-pENTR1A-F/R (Table S2) with a restriction site to generate the pENTR1A-CmJAZ1-likeΔJas vector. The pENTR1A-CmJAZ1-likeΔJas construct was then recombined with the pMDC43 vector via an LR Clonase™ II (Invitrogen) reaction to obtain the plant expression vector pMDC43-CmJAZ1-likeΔJas. The construct *35S::CmJAZ1-like*Δ*Jas* was then transformed into competent *Agrobacterium EHA105* cells for genetic transformation of chrysanthemum. Then, the *Agrobacterium* was used to infect the leaf discs to complete the transformation^30^. Young leaves of ‘Jinba’ tissue culture plantlets aged 30 to 35 days were selected and cut into leaf discs of 0.3 cm × 0.3 cm with wounds on the edges. Seedlings differentiated from infected leaf discs were transferred to MS medium containing 8 mg/L hygromycin for resistance screening. The forward primer GFP-F for the vector and the reverse primer for the ORF of the *CmJAZ1-like* gene (Table S2) were adopted to detect positive transgenic plants using RT-PCR. Subsequently, qRT-PCR was performed to assess the relative expression levels of the positive lines with the CmJAZ1-RT-F/R (Table S2) primers. Each sample was analyzed with three biological and three technical replicates. The wild-type and transgenic plants were cultivated in a greenhouse to observe their phenotypes, and each line contained 40 plants.

### RNA-Seq analysis

The seventh unfolded leaf of *CmJAZ1*Δ*Jas*-OX-#3 and wild-type plants were sampled 64 days after transplantation. Each sample contained three biological replicates. The total RNA from snap-frozen samples was extracted using an RNA Isolation Kit (Waryong, Beijing, China) following the manufacturer’s protocol and then subjected to Illumina sequencing at Beijing Genomics Institute (Shenzhen, China) using a BGISEQ-500 platform. Reads with low quality, joint contamination, and high content of unknown bases (N) were filtered out from the original data to obtain clean reads^55^. The Trinity program was then utilized to conduct de novo assembly of clean reads, and Tgicl was used to cluster the assembled transcripts for redundancy to obtain unigenes^56^. The assembled unigenes were annotated using seven functional databases (KEGG, GO, NR, NT, SwissProt, Pfam, and KOG). We then used Bowtie2 to align clean reads to the reference gene sequence and RSEM to calculate the expression levels of the genes and transcripts^57^. A Q-value below 0.05 was regarded as the threshold for DEGs^58^. Kyoto Encyclopedia of Genes and Genomes (KEGG) enrichment analyses of the annotated DEGs were performed on the BGI Interactive Reporting System (https://report.bgi.com/ps/login/login.html).

### Quantitative RT-PCR analysis

For the expression profile analysis of *CmJAZ1-like* in different tissues, young adventitious roots, stems, leaves, and shoot apexes were harvested at the vegetative stage, and the outermost whorl of ray florets was collected at the reproductive stage. The chrysanthemum cultivar ‘Jinba’ used for sampling was cultivated in a greenhouse under natural light conditions. To verify the expression of DEGs excavated by transcriptome sequencing and related to flowering time between the transgenic and wild-type plants, the seventh unfolded leaf of *CmJAZ1*Δ*Jas*-OX-3#, *CmJAZ1*Δ*Jas*-OX-1#, *CmJAZ1*Δ*Jas*-OX-2#, and wild-type plants was sampled 64 days after transplantation. Each sample had three replicates, and total RNA was extracted using an RNA Isolation Kit (Waryong) as mentioned above. Subsequently, 1 µg of total RNA was reverse transcribed using M-MLV reverse transcriptase (Takara Bio). qRT-PCRs (20 mL reaction mixture containing 10 μL of SYBR Premix Ex Taq™ II (Takara Bio)) were performed using a Roche LightCycler 96 real-time fluorescence quantitative PCR instrument (Roche, Basel, Switzerland). Primers used for qRT-PCR were designed online (https://www.ncbi.nlm.nih.gov/tools/primer-blast/) and are listed in Table S2. The chrysanthemum *elongation factor 1-alpha* (*EF1α*) gene (GenBank: AB548817.1)^59^ was chosen as the reference. Each sample was evaluated using three biological and three technical replicates. The relative abundance of transcripts was analyzed using the 2^−ΔΔCt^ method^60^.

### Statistical analysis

Significant differences among the different tissues were obtained by Duncan’s multiple-range test (*P* < 0.05). Significant differences between the transgenic and wild-type plants were determined using Student’s *t* test. *P < 0.05; **P < 0.01. All statistical analyses were conducted using SPSS v19.0 (SPSS Inc., Chicago, IL).

## Supplementary information

Supplementary Figure

Supplementary Table
