# Supplementary material for: Overexpression of the CmJAZ1-like gene delays flowering in Chrysanthemum morifolium
Source: Hortic Res. 2021 Apr 1;8:87. doi: 10.1038/s41438-021-00525-y (PMC8016864; doi:10.1038/s41438-021-00525-y)
Supplement: Supplementary file 1 — Supplementary Figure [file 41438_2021_525_MOESM1_ESM.docx]

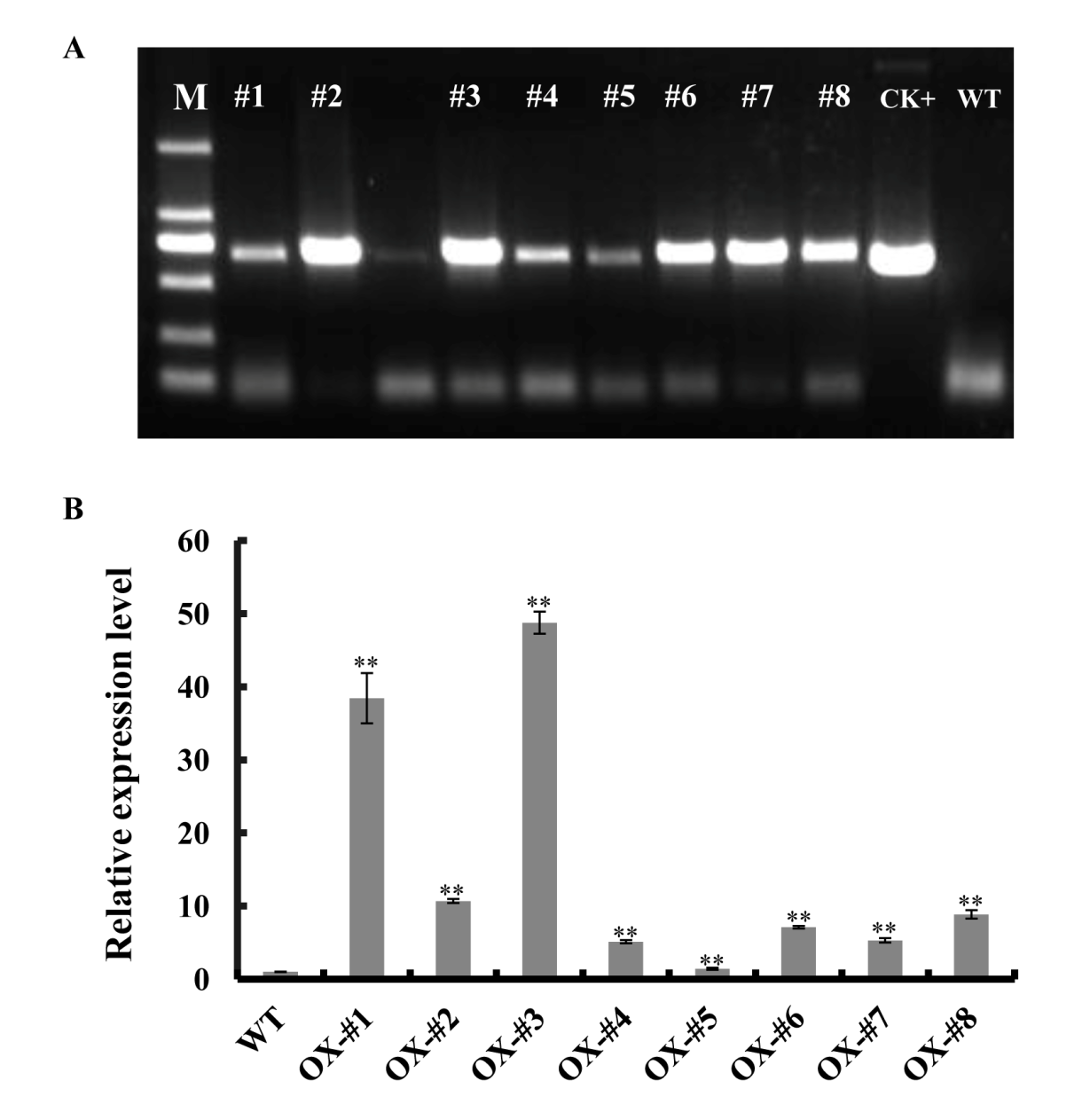


**Fig. S1 Analyses of *CmJAZ1-like△Jas*-overexpressing transgenic lines.** (A) PCR analyses of *CmJAZ1-like△Jas* transgenic lines at the DNA level. M: DL2000; #1-#8: Transgenic lines; CK+: Positive control; WT: Wild-type ‘Jinba’. (B) Relative expression levels of *CmJAZ1* in WT and OX transgenic lines. Error bars indicate SE (n = 3). Significant differences were determined by Student's t-test (***P* < 0.01).
